# A Novel Approach to Realizing Routine HIV Screening and Enhancing Linkage to Care in the United States: Protocol of the FOCUS Program and Early Results

**DOI:** 10.2196/resprot.3378

**Published:** 2014-07-31

**Authors:** Travis H Sanchez, Patrick S Sullivan, Richard E Rothman, Emily H Brown, Lisa K Fitzpatrick, Angela F Wood, Paloma I Hernandez, Amy S Nunn, Martin L Serota, Lisa Moreno-Walton

**Affiliations:** ^1^Emory UniversityAtlanta, GAUnited States; ^2^Johns Hopkins UniversityBaltimore, MDUnited States; ^3^George Washington UniversityWashington, DCUnited States; ^4^Family Medical Counseling ServicesWashington, DCUnited States; ^5^Urban Health PlanNew York City, NYUnited States; ^6^Brown UniversityPhiladelphia, PAUnited States; ^7^AltaMed Health ServicesLos Angeles, CAUnited States; ^8^Louisiana State University Health Sciences CenterNew Orleans, LAUnited States

**Keywords:** HIV, routine screening, testing, linkage

## Abstract

**Background:**

The United States health care system remains far from implementing the Centers for Disease Control and Prevention's recommendation of routine human immunodeficiency virus (HIV) screening as part of health care for adults. Although consensus for the importance of screening has grown, innovations in implementing routine screening are still lacking. HIV on the Frontlines of Communities in the United States (FOCUS) was launched in 2010 to provide an environment for testing innovative approaches to routine HIV screening and linkage to care.

**Objective:**

The strategy of the FOCUS program was to develop models that maximize the use of information systems, fully integrate HIV screening into clinical practice, transform basic perceptions about routine HIV screening, and capitalize on emerging technologies in health care settings and laboratories.

**Methods:**

In 10 of the most highly impacted cities, the FOCUS program supports 153 partnerships to increase routine HIV screening in clinical and community settings.

**Results:**

From program launch in 2010 through October 2013, the partnerships have resulted in a total of 799,573 HIV tests and 0.68% (5425/799,573) tested positive.

**Conclusions:**

The FOCUS program is a unique model that will identify best practices for HIV screening and linkage to care.

## Introduction

Nearly 8 years after the Centers for Disease Control and Prevention (CDC) recommended routine human immunodeficiency virus (HIV) screening for all Americans aged 13-64, the US health care system continues to struggle to make progress in meeting this important public health recommendation [1]. The scientific evidence supporting early diagnosis as a best practice has only become stronger [2,3]. In particular, the Cohen et al [2] study showed a greater than 90% reduction in HIV transmission to sex partners with earlier initiation of antiretroviral treatment. The Mugavero et al [3] paper emphasized how HIV testing is the prerequisite step into the continuum of sustained care and positive health outcomes for those living with HIV. Of persons in the United States who are living with HIV infection, 18% have not yet been diagnosed and one-third of those with a diagnosis are diagnosed late in the course of their disease [4,5]. Identifying innovative and robust approaches to reaching the large numbers of undiagnosed people living with HIV and doing so in a way that reaches them earlier in the course of their disease is key to making progress toward achieving viral suppression and providing its attendant clinical and prevention benefits. These approaches will not only need to substantially transform how we conduct HIV screening in clinical settings, but will need to address how the community as a whole responds to HIV screening and care.

There has been a steady accumulation of evidence and opinion that routine HIV screening is necessary to achieve the best individual and public health outcomes. A series of cost analyses beginning in 2005 suggested that routine HIV screening every 3 to 5 years would be justified for all but the lowest risk US adults [6-9]. Most recently, the United States Preventive Services Task Force (USPSTF) gave routine HIV screening a grade A recommendation [10].

**Figure 1:**
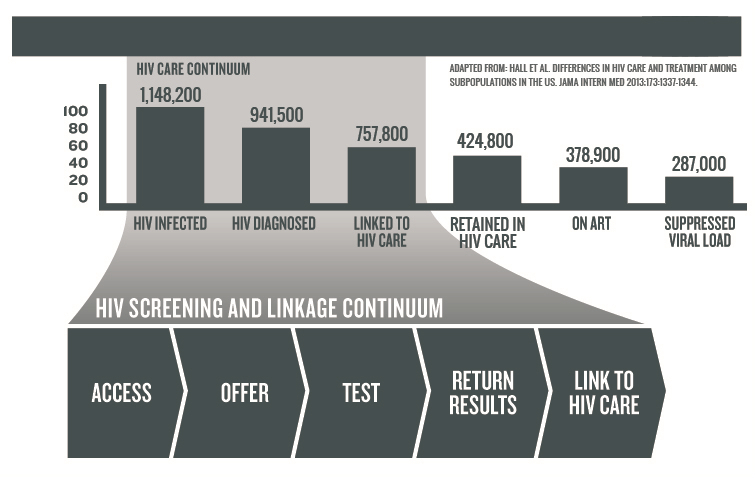
Routine HIV screening and linkage to care.

The HIV care continuum from diagnosis to successful engagement with medical care to viral suppression is now an important conceptual framework guiding HIV prevention and care efforts in the United States [3,4,11]. We propose extending the concept of a care continuum to an HIV screening and linkage continuum. Using the framework allows us to describe and then act on barriers and facilitators in a series of steps necessary for routine HIV screening to occur (Figure 1).

Broadly, the screening and linkage to care components of the cascade require that people have access to and are offered HIV screening, that they get tested and receive the results of their test, and that they have a reliable link to HIV care. Appreciation of these essential elements provides a framework to address barriers and facilitators to the introduction and expansion of HIV screening in both clinical and community settings. The barriers may include lack of knowledge regarding routine screening recommendations, lack of awareness of community epidemiology of HIV, concerns about regulatory requirements for consent and pretest counseling, uncertainty regarding reimbursement, competing priorities for time, stigma associated with HIV and HIV testing and concerns about how people will respond to the offer of an HIV test [12-21]. The facilitators for routine screening in clinical and community settings include better recommendations, regulations, and health policies. Most states have already made considerable progress in eliminating or reducing pretest counseling requirements [22]. Expanded health care coverage that will be implemented through the Affordable Care Act is likely to increase the probability of a care visit [23]. The USPSTF grade A recommendation for HIV screening clears the way for reimbursement from all medical insurance plans eventually decreasing provider and patient concerns about reimbursement.

During this period of regulation and policy change, a major opportunity exists to develop and validate new approaches for improving routine HIV screening and linkage to care practices in the United States. This will require adopting new program models across multiple types of settings in the clinics and communities in cities that are most highly impacted by HIV. The amount of HIV screening in most clinical settings needs to increase by orders of magnitude to reach the standard of care set by the CDC and USPSTF recommendations. Additionally, not everyone routinely accesses medical care, including those who have had a previous HIV diagnosis. There remains a role for better community-delivered routine HIV screening and linkage to care coordination models. To accomplish such transformational changes in our clinical and community HIV screening programs, new approaches must be designed, efficiently evaluated, and disseminated. Promoting broader uptake will require closer to real-time sharing of these models to allow a rapid pace of knowledge application. This paper describes the overall approach of a large-scale and multi-component program that is specifically designed to address these needs.

## Methods

### FOCUS Program Overview

**Figure 2:**
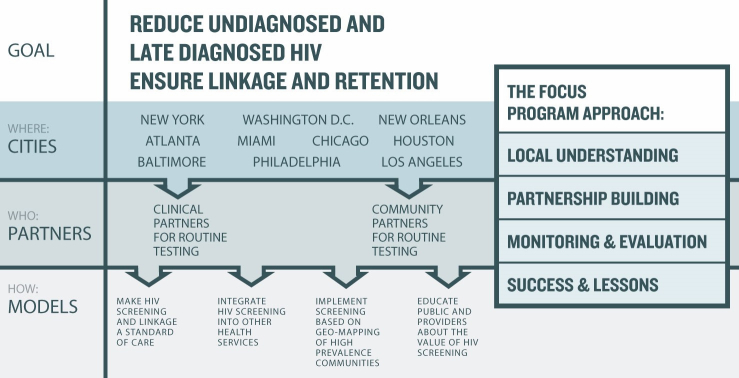
FOCUS strategy.

HIV on the Frontlines of Communities in the United States (FOCUS) was launched in 2010 and is a program of Gilead Sciences, Inc. The overall program goals are to create, implement, improve, and rapidly disseminate transformative approaches that make HIV screening a truly routine practice in both clinical and community settings and that improve linkage to HIV care for all persons living with HIV. The FOCUS program is implemented through a multilevel strategy (Figure 2).

The first level is “where” the FOCUS program is implemented: cities in the United States and communities within those cities that are most highly impacted by HIV infection. The second level is "who" implements the FOCUS program: partners within health departments and from clinical or community-based organizations who can be leaders of change in these cities. The third level is “what” should be implemented: model projects implementing components of the FOCUS program that are customized to their settings and all with the same overall FOCUS program goals. The FOCUS program uses a consistent approach to plan and coordinate activities by: building strategic partnerships, conducting program monitoring and evaluation, and communicating program successes and lessons learned through informal and traditional mechanisms.

Working with a coalition of local health care and community leaders, a FOCUS Regional Lead in each city conducts a comprehensive assessment of the context for the program, translates the overall program model into city-wide plans of action, and builds strategic clinical and community partnerships that implement those plans (Multimedia Appendix 1). Partners with the potential to achieve FOCUS goals and who are leaders of broader change in their fields are invited to propose projects. The median project budget is approximately US$175,000 for projects involving discrete activities that are implemented in a 12-month period. Funds support electronic medical records (EMR) modifications, data management, continuous quality improvement (CQI), personnel for linkage to care, upgrades to laboratory equipment, and dissemination of findings. If initial project objectives are met and if new and more expansive activities are proposed, partners can receive additional funding in subsequent years. Each 12-month project for a partner is counted separately for purposes of this report. FOCUS projects are specifically funded to support approaches that will ultimately be sustainable through other public investment or by third party reimbursement, such as health insurance.

### FOCUS Data Collection, Monitoring, and Evaluation

All FOCUS partners report a common set of de-identified outcome indicators to a centralized database monthly (Multimedia Appendix 2). The requirement for standard and continuous data reporting produces a culture of data-driven decision making within FOCUS and among partners. The central data repository allows for quantitative comparisons between partner sites and project components that can be used to support continuous program improvement. Partners use the data in real-time and at facility-, unit-, or provider-level to continually monitor progress toward routine provision of screening, and modify their activities to achieve better results. Regional Leads use the de-identified data to monitor partner successes and support partners in making program improvements.

The FOCUS program uses an iterative process in which lessons learned from one partnership can be immediately applied to other similar partnerships, producing a real-time learning environment. This is facilitated by the frequency of outcome monitoring, the diversity of partnerships and the flexibility of the funding mechanism. Regional Leads facilitate the sharing of successful project components throughout similar settings and partnerships in their cities. Communication and networking among Regional Leads allows lessons learned in one city to be disseminated between cities. FOCUS uses program monitoring information to help set priorities for future partnerships and transform practices across existing partnerships. FOCUS partners also develop individual plans to disseminate lessons learned to others in their fields.

## Results

### Program Overview

**Figure 3:**
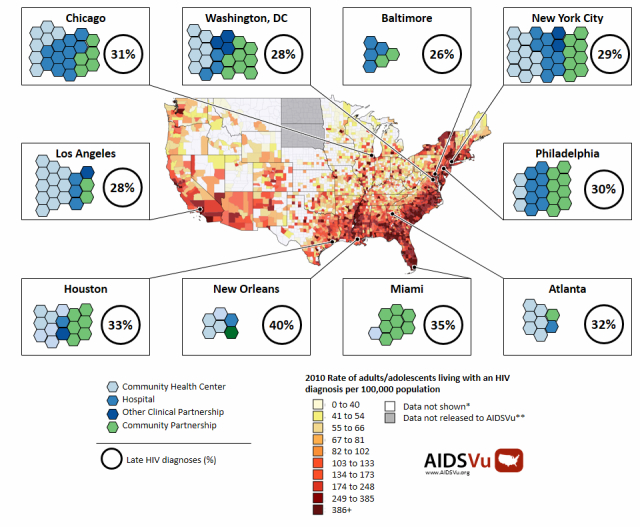
FOCUS partnerships by city.

The FOCUS program has been implemented in Atlanta, Baltimore, Chicago, Los Angeles, Houston, Miami, New Orleans, New York City, Philadelphia, and Washington, DC (Figure 3). These 10 cities represent 40% of the prevalent HIV diagnoses in the entire United States through 2010, and 36% of the new HIV diagnoses made in 2011 [24]. These cities also all have communities within them where greater than 1 out of every 50 people is living with HIV. Consistent with the national average, about 30% were diagnosed with HIV infection late in the course of their illness [25]. From program launch in 2010 through October 2013, 153 partnerships have been developed in the 10 cities. FOCUS partnerships have resulted in 799,573 HIV tests being conducted with 0.68% (5425/799,573) persons testing positive. These partnerships are of two types: clinical partnerships and community partnerships.

### Clinical Partnerships

#### Overview

**Figure 4:**
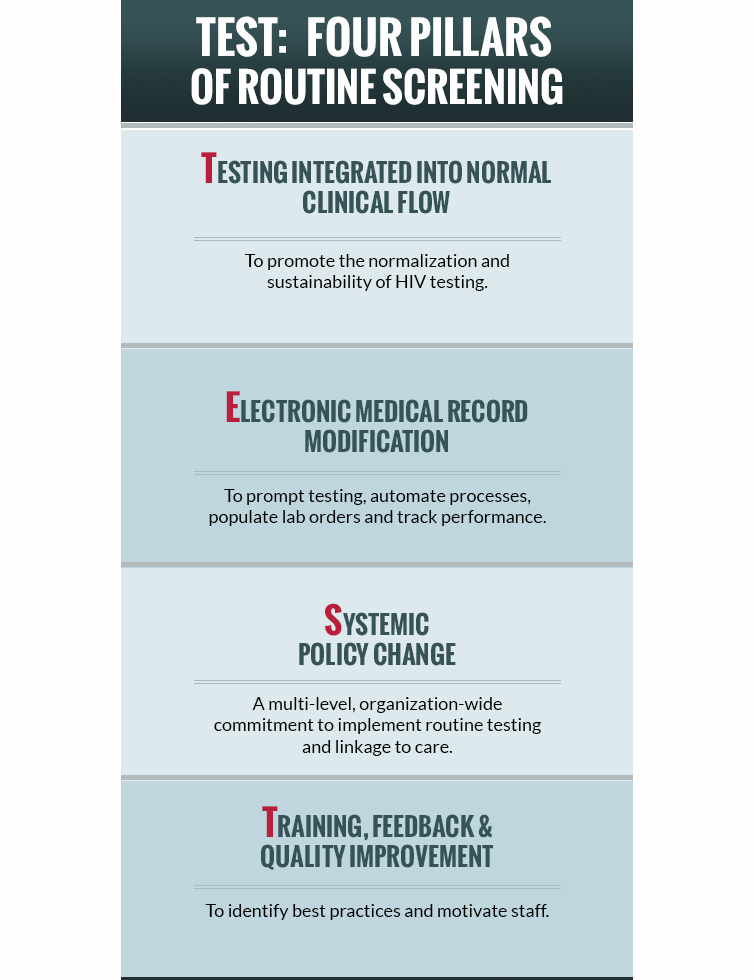
TEST: four pillars of routine HIV screening in clinical settings.

All partnerships in clinical settings use the following components. We call these the “four pillars of routine HIV screening” (Figure 4 and Table 1).

**Table 1:** Characteristics of FOCUS partnerships, 2010-2013.

Partnership setting^a^		Number of partnerships (%)	Cities^b^	Project components
**Clinical**				
	Community health centers	57 (37)	ATL, CHI, HOU, LA, MIA, NOLA, NYC, PHI, DC	• Testing integrated into normal clinic flow • Electronic medical record modification • Systemic policy change • Training, feedback, and quality improvement
	Hospitals	40 (26)	ATL, BAL, CHI, HOU, LA, NOLA, NYC, PHI, DC	
	Other clinics	7 (5)	HOU, LA, NYC, DC	
**Community**		49 (32)	ATL, BAL, CHI, HOU, LA, MIA, NOLA, NYC, PHI, DC	• Community-delivered screening • Public and provider education • Community-led linkage to care
TOTAL		153		

^a^Partnership types are defined as follows: Community health centers - provide comprehensive primary care, either grant-supported federally qualified health centers or non-grant-supported health centers certified by the Health Resources and Services Administration and the Centers for Medicare and Medicaid Services; Hospitals - provide general and specialized medical, surgical, or mental health services, can be on an inpatient or outpatient basis, and can be governmental, academic, and/or private institutions; Other Clinics - provide clinical services to patients but are not classified as health centers or hospitals, includes sexually transmitted disease, general wellness, and family planning clinics; and Community Partnerships - organization conducting activities outside of the clinical setting and in the community, includes community-based organizations, health centers, health departments, and academic institutions

^b^City Abbreviations: ATL - Atlanta, BAL - Baltimore, CHI - Chicago, HOU - Houston, LA - Los Angeles, MIA - Miami, NOLA - New Orleans, NYC - New York City, PHI - Philadelphia, DC - Washington DC

#### Testing Integrated Into Normal Clinic Flow

The best efficiencies for HIV screening in clinical settings are likely to be gained when testing is fully integrated into clinic flow. These changes not only reinforce provider perceptions that HIV screening is a routine part of care, but also improve the efficiency of the test offer. FOCUS partners develop ways to incorporate the offer and administration of the test into patient intake or triage processes. Missed opportunities for HIV diagnosis also continue to exist in other clinical settings where routine HIV screening should be available, such as at sexually transmitted disease and family planning clinics. The challenge has been ensuring seamless integration of HIV screening into these other health services. Rather than staffing and processes that make HIV screening something exceptional from other services offered, the same staff use existing infrastructure to conduct all of the health screening activities at these facilities. This is accomplished through simplified processes for making the test offer, obtaining appropriate documentation, collecting specimens, and conducting the HIV test. Full integration of the HIV test into normal clinical flow also increases the likelihood of long-term sustainability of HIV screening as it maximizes the use of indigenous clinic staff rather than staff dedicated solely to HIV testing. Some partners are using dedicated staff to help patients who test positive get linked to HIV care. Partners are implementing these changes through multiple methods such as standing physician orders for an HIV test, automating EMR reminders to offer/order a test, creating staff fact sheets and checklists for HIV screening, and conducting training with indigenous staff.

#### Electronic Medical Record Modification

EMR and laboratory information systems are now standard medical practice. Ensuring that HIV screening is fully integrated into these settings requires changes to the systems already in use. Modifications to these systems use a variety of approaches to integrate the test offer and administration. Partners use these systems to create an algorithm to determine eligibility for the test offer, prompt staff to make offers, order HIV laboratory tests, record results, conduct CQI and support billing. Some systems have also been modified to trigger multiple opportunities to make the offer for HIV testing either at the same visit or in successive visits.

#### Systemic Policy Change

A critical step in making systemic improvements to HIV screening programs in clinical settings is changing perceptions of key leadership, such as the chief executive officer, chief medical officer, chief nursing officer, laboratory director, chief operating officer, and chief of risk management. Unlike models that offer specialized or targeted HIV testing and linkage to care services, integration into routine clinical practice requires an organization-wide commitment from leadership and clinic staff, and involves a continuous process of uncovering barriers and developing solutions to change the perception of HIV testing from being a specialized service to a routine one. In addition, FOCUS partners are establishing improved policies and procedures for ensuring that diagnosed persons are effectively linked to HIV care. Partnerships are engaging with leadership and clinic staff through multiple methods, such as policy briefs, baseline data assessments, cost analyses, leadership meetings, and provider champions.

#### Training, Feedback, and Quality Improvement

Systematic program improvement relies on collecting and effectively using key indicators to continuously monitor progress and outcomes. The use of data to make program improvements is especially important for implementation of routine HIV screening programs in clinical settings. To ensure that HIV screening is being implemented in a truly routine manner, these systems must be able to track and monitor unique patient visits, eligibility for testing, test offers, tests conducted and linkage to care for those identified as positive. All clinical partners are required to have a monitoring and feedback system that tracks these elements as an integral part of their program. These CQI systems allow partners not only to collect monitoring data, but incorporate plans for routinely sharing information with clinic leadership, providers, and staff. An integral part of these projects is to also institute staff and provider training, incorporating feedback from the monitoring systems. When opportunities for improvement are evident, the CQI process uses detailed plans of action to make improvements. The ultimate purpose of CQI is to promote full scale-up and sustainability in these settings.

In addition to the four pillars of routine screening, partners are also encouraged to critically consider the type of HIV test that they employ. HIV test technologies have been continually evolving, serologic assays are becoming increasingly sensitive, and new testing platforms have been developed that allow for large throughput of samples in the laboratory. This may be particularly important in HIV screening programs in clinical settings that not only require a rapid return of a test result but may also be challenged with large numbers of HIV tests. These large-scale HIV screening platforms allow better integration into the routine laboratory practices of large-volume medical facilities by using blood specimens that are already being collected for other purposes. The newest generation of these platforms also have the added benefits of using 4^th^ generation HIV serologic assays that are compliant with new HIV screening algorithms for laboratories and detect HIV infection earlier than most other point-of-care rapid tests and laboratory-based tests [26,27]. They also allow confirmation of an HIV diagnosis to be made in a single visit, which may facilitate more timely linkage to HIV care. Several partnerships in large-volume clinical settings are just now implementing these new test technologies to improve routine HIV screening.

Partnerships in clinical settings include 57 community health centers in 9 FOCUS cities (Table 1). These community health centers have conducted 257,869 HIV tests and had 0.54% (1398/257,869) of persons test positive (Table 2). Partnerships in clinical settings also include 40 hospital systems in 9 cities. These hospitals have conducted 385,667 tests and had 0.72% (2793/385,667) of persons test positive. The program has also built partnerships in other clinic settings that integrate HIV screening and linkage to care into other health services through 7 partnerships in 4 cities. These other clinics have conducted 54,798 tests and had 0.52% (286/54,798) of persons test positive. Using data to support project improvements between and within partnerships is especially important in identifying and replicating aspects of routine screening in clinical settings that are most effective at reaching interim program goals. An example of this iterative improvement process for FOCUS clinical partnerships is presented in Textbox 1. In addition to sharing lessons within the FOCUS program, partners in clinical settings have also begun to disseminate project outcomes and details of their individual models. Clinical partnerships have resulted in 15 conference presentations and 1 journal publication about their projects [28-43].

**Table 2:** FOCUS testing outcomes, 2010-2013.

Partnership setting			Number of tests		Number of persons tested positive		% of persons positive			Partner presentations, publications, and media
**Clinical**										
	Community health centers		257,869		1398		0.54			28-34, 43
	Hospitals		385,667		2793		0.72			35-39, 43
	Other clinics		54,798		286		0.52			40-42
**Community**			101,239		948		0.94			44-54
Total			799,573		5425		0.68

**Iterative improvement and dissemination in clinical settings.**

The iterative improvement process is best illustrated by two early FOCUS partnerships to implement routine HIV screening in large, multifacility, community health centers. AltaMed Health Services based in Los Angeles implemented first and learned early on that success depended upon the engagement of executive leadership through a CQI process. Leadership authorized modifications to the EMR to designate the eligible patient population, prompt offers, and track tests. This resulted in a substantial increase in HIV testing at AltaMed. They were also able to demonstrate improvements in HIV testing after implementation of mandatory staff training about routine HIV screening. Some months later, Urban Health Plan in New York City adapted its already well-developed CQI program to also address routine HIV screening. Drawing upon AltaMed’s experience, Urban Health Plan modified the EMR and tracked offers and tests not only by facility but also by provider. Provider-level tracking and a weekly review of results identified good outcomes as well as pockets of resistance, which were then addressed through provider-level coaching about routine HIV screening. Both partnerships used their CQI data to demonstrate that the desired scale and normalization of HIV testing was best achieved through laboratory-based testing integrated into standard clinical practices. These processes, methods, and tools were shared with all FOCUS partnerships in clinical settings and were further adapted and refined. This program model is now institutionalized for FOCUS as "TEST: Four Pillars of Routine Screening," and is incorporated into all new proposals for partnerships in clinical settings.

### Community Partnerships

#### Overview

Partnerships in community settings employ varying combinations of activities related to routinely screening persons for HIV infection and ensuring linkages to HIV care. Partnerships implemened the following components.

#### Community-Delivered HIV Screening

HIV testing opportunities in community settings not only reach persons who are not engaged with health care, but can also reduce stigma and change attitudes regarding HIV and HIV testing in all settings. Partners employing this model use geographical mapping tools such as AIDSVu.org and local HIV program data to identify communities that are most highly impacted by HIV and identify venues for community-delivered HIV screening. Unlike many other outreach testing projects that conduct more targeted HIV testing, FOCUS partners offer a test to everyone encountered in these settings. These projects use a number of innovative approaches, such as retail-outlet and pharmacy testing, testing at high-volume public service centers like department of motor vehicle offices, and door-to-door testing in the neighborhoods targeted through epidemiologic mapping. An example of a community-delivered HIV screening project is presented in Textbox 2 [44,45].

**Community-delivered HIV screening.**

In October 2010, Family Medical Counseling Services, Inc implemented a novel HIV testing strategy at the Department of Motor Vehicles (DMV) in a highly impacted area of Washington DC. This DMV office provides driver’s license and automobile tag services to over 150,000 residents annually. Dedicated project staff introduce the opportunity to test to the entire waiting room at frequent intervals and discuss the importance of routine HIV testing. Everyone awaiting DMV services is offered a test. Rapid HIV testing is fully integrated into the flow of the DMV visit usually while people are waiting for services, is conducted in a private office inside the DMV, and all who test positive are immediately referred to care and support services. In 2011, this testing strategy was expanded to the Income Maintenance Center (IMC), the government office that provides residents with public benefits including food stamps, financial assistance, and health insurance services. The project has the primary goals of promoting and increasing access to HIV testing among residents in the high prevalence area east of the Anacostia River in Southeast, DC. Important lessons learned from these programs is that it is acceptable, feasible, and sustainable to implement a fully integrated HIV screening program in a high volume public services office if you work closely with service center staff, there is adequate space available to conduct confidential testing, and the duration of the service center visit will enable time for the entire HIV screening process. Through October 2013, the DMV and IMC screening program has conducted 25,361 HIV tests and had 0.56% (143/25,361) of persons test positive.

#### Community-Led Linkage to Care Coordination System

HIV screening programs have traditionally relied on specialized linkage to care service providers or passive referrals to HIV care and treatment. FOCUS partners have made multiple improvements in their linkage systems by adopting active approaches to link people to care and treatment. Though all partners are required to make improvements in linkage to care processes for people who test positive in their own programs, some novel FOCUS community partnerships act as coordinators for linkage to care activities for everyone who tests positive in their communities. These projects coordinate linkages from multiple places that do HIV testing and to multiple places that provide HIV care in the community. These projects include providing assistance to schedule the first medical care appointment, obtaining better contact information to allow ongoing support of diagnosed persons, ensuring that the first and subsequent medical appointments were attended, and establishing more effective collaborations with infectious disease or HIV primary care providers.

#### Public and Health Care Provider Education about HIV and HIV Testing

To reduce stigma and normalize attitudes regarding HIV and HIV testing, some community partners implement projects specifically designed to educate patients or providers about HIV. Academic partners are developing and delivering curriculum and practical experiences in HIV screening for students and midcareer health care professionals, including physicians, nurses, dentists, and pharmacists. Health departments are creating and disseminating tools to help health care providers understand HIV screening laws and to simplify offers, testing, and interpretation of test results. Other partners are producing information campaigns with messages about the availability of HIV screening options, and reinforcing messages that HIV infection in these highly impacted communities is just as much about where you live as it is about what you do (ie, not just about risk-based HIV screening).

The FOCUS program engages 49 community partnerships across all 10 cities to offer HIV screening in unique settings and support innovative stakeholder engagement and public education campaigns (Table 1). Organizations conducting HIV screening in the community have conducted 101,239 tests and had 0.94% (948, 101,239) persons test positive (Table 2). FOCUS partnerships in community settings have resulted in 10 conference presentations and 1 peer-reviewed journal article [44-54].

## Discussion

### Improvements in Routine HIV Screening are Needed

HIV transmission continues at a steady rate in the United States during an era of new testing guidelines, effective HIV prevention strategies, and advances in care and treatment: an estimated 48,000 people in the United States are newly infected each year [55]. Approximately 200,000 people living with HIV are not yet diagnosed [4]. The scope of the linkage and retention in care problem is also large, with approximately 500,000 people living with HIV in the United States who know they are infected, but who are not currently accessing HIV medical care [4]. Despite strong and consistent recommendations for routine HIV screening as part of medical care in the United States [1,10], routine screening is not yet a realized standard of care in most clinical settings. In the first 5 years after the CDC recommendation for routine HIV screening in clinical settings there have been statistically significant but practicably insufficient increases in HIV testing; still more than 50% of adults have never had an HIV test [56]. The rate of ever having an HIV test only grew 9% from 2000 to 2010. By sustaining this level of change, it would take another 40 years to achieve testing rates of more than 80%. The President of the United States has identified an urgent need for a renewed focus on both HIV screening and linkage to care programs [11].

### Current Implementation of FOCUS

The FOCUS program is attempting to address these needs by being a catalyst and proving ground for these transformational changes. The FOCUS program has established 153 partnerships in 10 highly impacted US cities, resulting in almost 800,000 HIV tests and more than 5400 HIV diagnoses. More detailed and comprehensive program evaluation activities are still underway, but clinical and community partners are already producing interim findings and project component details in the form of media stories, conference presentations, and peer-reviewed papers.

FOCUS partnerships are creating models that are intended to be practical to implement, be sustainable in the long term, and be replicable in similar settings. It should not be discounted that early experiences highlight the broad scope of changes and the intensive resources needed: fully integrating HIV screening into clinic flow, improving provider training, altering public perceptions of HIV screening, appropriately using new HIV testing technologies, maximizing the potential of EMRs, streamlining public laws and policies, and reducing stigma toward those living with HIV. All of these require significant investment of human and infrastructure resources to bring about sustainable change.

The FOCUS program fills a gap in existing funding mechanisms and sources. FOCUS partners are more than demonstration projects to illustrate improved incremental approaches to implementing routine HIV screening. Previous demonstration projects often resulted in improvements in local practices, but dissemination and propagation of best practices was less predictable. The FOCUS program includes mechanisms for rapid feedback and sharing of best practices, increasing the impact of lessons learned by promoting timely dissemination to other partners. The FOCUS program is also different from implementation science by nature of the quick cycles of implementation, evaluation, and change. Plans are underway to conduct formal program evaluations and create toolkits based on FOCUS project components. These evaluations and toolkits will also help affect broader systems change for routine HIV screening and linkage to care.

### Future of FOCUS

The FOCUS program is a unique model for how nongovernmental funding sources can engage in dynamic and evidence-based projects to identify best practices for HIV screening and linkage to care. The FOCUS program will increase the volume of screening directly supported by its funding, but the primary goal is to find ways to transform existing systems and achieve sustainable change through reducing barriers and capitalizing on innate system strengths. The ultimate success of the FOCUS program will lie in the commitment to an overall vision of excellence, data-driven decision making, a shared learning environment, and a culture of continually seeking transformative innovation. Importantly, the success of the program will rely upon the high level of commitment and expertise of the wide variety of FOCUS partners on the frontlines of the US HIV epidemic every day.

## Multimedia Appendix 1

FOCUS Plan of Action Template.

## Multimedia Appendix 2

FOCUS Monitoring Indicators.

## Abbreviations

CDC: Centers for Disease Control and Prevention
CQI: continuous quality improvement
DMV: department of motor vehicles
EMR: electronic medical records
FOCUS: Frontlines of Communities in the United States
HIV: human immunodeficiency virus
IMC: income maintenance center
USPSTF: United States Preventive Services Task Force
