# Supplementary material for: A Novel Approach to Realizing Routine HIV Screening and Enhancing Linkage to Care in the United States: Protocol of the FOCUS Program and Early Results
Source: JMIR Res Protoc. 2014 Jul 31;3(3):e39. doi: 10.2196/resprot.3378 (PMC4129189; doi:10.2196/resprot.3378)
Supplement: Supplementary file 1 [file resprot_v3i3e39_app1.pdf]

# Appendix 1: FOCUS Plan of Action Template

**Frontlines of Communities in the United States (FOCUS)**

**PLAN OF ACTION**

***Frontlines of Communities in the United States*** **(FOCUS)**

**Table of Contents**


I. FOCUS Overview

II. Overview of City/Region

III. Epidemiological Profile of City/Region

IV. HIV/AIDS Prevention and Control in City/Region

V. Reaching the Undiagnosed: City/Regional Public Health Framework for FOCUS

VI. Plan of Action to support FOCUS Goals 2012-2013

VII. Program Models

VIII. Attachments & Appendices

## I. Focus Overview

### Frontlines of Communities

FOCUS is designed to work with community, health and hospital systems, health providers, civic leaders, advocates and policymakers in the quest for sustainable solutions to the nation's HIV/AIDS epidemic. A limited number of partners are selected in geographic areas or communities acutely impacted by HIV/AIDS epidemic. Partners include local health departments, , hospitals, community health centers/community clinics, STI clinics, community organizations and others. FOCUS is grounded in the belief that a comprehensive local approach, which engages partners at every level and is focused on systems change and long-term program sustainability, is the best way to reach the undiagnosed and ultimately decrease the rate of new infections.

FOCUS promotes and supports evidence-based best practices. By making community-driven investments that build upon and challenge experiences and successes of our partners, FOCUS seeks to improve the work being done to reverse the HIV/AIDS epidemic. Wrapped around these investments are policy and reform goals that support the 2006 Centers for Disease Control recommendations on routine opt-out HIV/AIDS testing.

### FOCUS Goals

- Reduce the number of undiagnosed individuals and link them to care
- Decrease the number of late diagnoses of HIV infection
- Make routine HIV screening a standard of care within healthcare settings
- Normalize the HIV testing process

### FOCUS Objectives

- Identify healthcare settings to implement and monitor routine opt-out screening in an efficient manner and non-burdensome manner so that it is accepted by providers and patients
- Reach a greater proportion of the undiagnosed in FOCUS targeted cities by supporting innovative and best practice screening and linkage to care programs in non-traditional settings
- In collaboration with local Departments of Health work to develop more accurate localized measurements of the number

of undiagnosed in targeted cities by race/ethnicity, age and gender as legally available
- Identify and amplify new voices by empowering non-traditional change agents and local champions
- Generate greater modern day awareness of HIV/AIDS by highlighting partners and their success and challenges via a comprehensive communications/publications strategy

## II. General Overview of City or Region

- Racial/ethnic breakdown
- Socioeconomic breakdown, poverty and disparities
- LGBTQ population information
- Other relevant demography
- Local economy
- Incarceration data

## III. Epidemiological Profile: City/Regional Overview of HIV/AIDS

**[Local epidemiological data, charts and graphs]**

*Race and Ethnicity*

*Age*

*Other demographics & Risk Groups*

*Deaths*

*Late stage diagnosis*

*Testing trends*

*Geographic breakdown*

*Data tables*

## IV. HIV/AIDS Prevention and Control

**Prevention Efforts: State or Region**

- History and overview
- Public health jurisdiction
- Funding sources

- Local resources
- Leadership
- Political considerations
- Strategic changes

**Citywide HIV Prevention Efforts:**

a. Resources: List and summaries of relevant prevention programs and their funding sources:

- ASOs
- Public-private partnerships
- Health care providers
- MSM initiatives
- Women's initiatives
- IDU, syringe exchange programs
- Linkage to Care
- Social media marketing efforts

Other efforts

- Data sharing efforts
- Policy and legislation
- Other sectors (corrections, schools, etc.)

b. Coordinating bodies
  - Planning Councils

c. Additional efforts

## V. FOCUS Goals: Reaching the undiagnosed

1. Reduce the number of undiagnosed individuals and maintain high linkage to care
2. Decrease the number of late diagnosis of HIV infection
3. Make routine screening a standard of care
4. Normalize the testing process

| **Demographic Focus** | **Institutional & Community Focus** |
|---|---|
| ♦ Race<br>♦ Gender<br>♦ Age | ♦ Geographically mapped high prevalence communities |

| | |
|---|---|
| ♦ Other demography | ♦ Community Health Centers<br>♦ Public and Urban Hospitals<br>♦ Select Community Settings: STD Clinics, Charity Clinics, DMV |

## VI. PLAN OF ACTION to support FOCUS Goals 2012 - 2013

**Strategy 1:** [Example: *Enhance buy-in and capacity of routine opt-out testing in health care settings that serve low income, high prevalence communities*].

*Goal:* [Example*: Identify, inform, and engage health care providers on CDC recommended opt-out HIV testing as standard of care in health settings to address gaps in testing offers].*

*Objective: [*Example*: Engage 3 new high volume community health centers within high prevalence areas*]

**[Sample] Activities:**

- *Partner with medical associations whose members are strategically positioned in the community and have capacity to routinize HIV testing*
- *Identify clinical settings that serve target populations*
- *Assess opportunities to work with Federally Qualified Health Centers and primary health clinics*
- 
- *Assess knowledge and implementation of routine, opt-out testing*

**Strategy 2:** [Example: *Enhance capacity for routine HIV testing and linkage to care, aiming to target urban, high volume hospitals*]

*Goal 1* Increase the number of hospitals that incorporate testing into emergency care, primary care, and outpatient clinic services.

*Objective 1:* Add two high volume urban hospitals with the capacity to implement and routinize testing

**[Sample] Activities:**

- Assess opportunities to engage local urban hospital emergency departments for implementation of opt-out testing.
- Explore partnerships with local emergency departments to transition from counselor-based, intermittent testing to screening that is fully integrated as a normal part of ED flow. Assess and support the use of the most sensitive testing technologies
- Work with hospital leadership to assess gaps, needs and organizational system changes needed to implement routine screening. via

**Timeline for Partnership Development and Implementation**

## VII. ACTIVE PROGRAM MODELS

| **[Partner Name]**<br>**Target Communities:**<br>**Partnership Period:** | | |
|---|---|---|
| ***Project Model:***<br><br>***What is being tested?*** | ***Why Selected*** | ***Goals:***<br><br>***Progress:*** |

**CONCEPT STAGE:**

| **[Partner Name]**<br>**Target Communities:**<br>**Partnership Period:** | | |
|---|---|---|
| ***Project Model:***<br><br>***What is being tested?*** | ***Why Selected (DRC)*** | ***Goals:*** |

**Key Voices and Influencers**

| Contact Name | Title/Affiliation | Status | Notes |
|---|---|---|---|
| | | | |

| | | | |
|---|---|---|---|
| | | | |
| | | | |

FOCUS Advisory Board Members:

- [Name, Title, Affiliation]

VIII. **Attachments & Appendices**
