# Supplementary material for: A Novel Approach to Realizing Routine HIV Screening and Enhancing Linkage to Care in the United States: Protocol of the FOCUS Program and Early Results
Source: JMIR Res Protoc. 2014 Jul 31;3(3):e39. doi: 10.2196/resprot.3378 (PMC4129189; doi:10.2196/resprot.3378)
Supplement: Supplementary file 2 [file resprot_v3i3e39_app2.pdf]

Appendix 2: FOCUS Monitoring Indicators

All HIV Tests

- Client or Form ID (randomly generated unique number)
- Clinic/Site Name
- Patient Visits
- Patients Eligible for HIV Testing
- HIV Tests Offered
- HIV Tests Performed
- Declined HIV Screening
- Reasons for Declining HIV Screening (optional)
- Age Group
- Race and Ethnicity
- Gender
- Self-Reported Previous Test Result
- HIV Test Result
- Provision of HIV Test Result
- Newly Identified HIV Positive
- Previously Identified HIV Positive

HIV Positive Individuals Only

- Linkage to Care: First Appointment Kept
- Reasons First Appointment Not Kept
- Linkage to Care: Second Appointment Kept
- Possible Mode of HIV Transmission
- First CD4 Count within 3 Months of Diagnosis
- First HIV Viral Load within 3 Months of Diagnosis

Acute HIV Infection

(If applicable to project scope)

- Suspected Acute Infection
- Gender
- Age Group
- Race/Ethnicity

STD Tests

(If applicable to project scope)

- STD Tests Performed
  - Syphilis
  - Chlamydia
  - Gonorrhea
- Positive STD Test Results
  - Syphilis
  - Chlamydia
  - Gonorrhea
